# Spatiotemporal expression and regulation of peptidase inhibitor 3 and secretory leukocyte protease inhibitor at the maternal–fetal interface in pigs

**DOI:** 10.5713/ab.22.0415

**Published:** 2023-02-26

**Authors:** Soohyung Lee, Inkyu Yoo, Yugyeong Cheon, Hakhyun Ka

**Affiliations:** 1 Division of Biological Science and Technology, Yonsei University, Wonju 26493, Korea

**Keywords:** Endometrium, Peptidase Inhibitor 3, Pig, Pregnancy, Secretory Leukocyte Protease Inhibitor, Serine Protease Inhibitor

## Abstract

**Objective:**

Two serine protease inhibitors, peptidase inhibitor 3 (PI3) and secretory leukocyte protease inhibitor (SLPI), play important roles in protease inhibition and antimicrobial activity, but their expression, regulation, and function at the maternal–fetal interface in pigs are not fully understood. Therefore, we determined the expression and regulation of PI3 and SLPI in the endometrium throughout the estrous cycle and at the maternal–fetal interface in pigs.

**Methods:**

Endometrial tissues during the estrous cycle and pregnancy, conceptus tissues during early pregnancy, and chorioallantoic tissues during mid to late pregnancy were obtained, and the expression of *PI3* and *SLPI* was analyzed. The effects of the steroid hormones estradiol-17β (E2) and progesterone (P4) on the expression of *PI3* and *SLPI* were determined in endometrial explant cultures.

**Results:**

*PI3* and *SLPI* were expressed in the endometrium during the estrous cycle and pregnancy, with higher levels during mid to late pregnancy than during the estrous cycle and early pregnancy. Early-stage conceptuses and chorioallantoic tissues during mid to late pregnancy also expressed *PI3* and *SLPI*. PI3 protein and *SLPI* mRNA were primarily localized to endometrial epithelia. In endometrial explant cultures, the expression of *PI3* was induced by increasing doses of P4, and the expression of *SLPI* was induced by increasing doses of E2 and P4.

**Conclusion:**

These results suggest that the *PI3* and *SLPI* expressed in the endometrium and conceptus tissues play an important role in antimicrobial activity for fetal protection against potential pathogens and in blocking protease actions to allow epitheliochorial placenta formation.

## INTRODUCTION

The innate immune system is a first-line defense mechanism against infection and inflammation [1]. The innate immune system is composed of physical barriers, including tight junctions and the mucus layer of epithelium; cellular components, including natural killer cells, neutrophils, monocytes, and macrophages; and humoral components, including naturally occurring antibodies, complements, and antimicrobial peptides (AMPs) [2]. Among those innate immune system components, AMPs have inhibitory effects against bacteria, fungi, parasites, and viruses and include a class of small peptides that contains cathelicidins, α- and β-defensins, peptidase inhibitor 3 (PI3), secretory leukocyte protease inhibitor (SLPI), and the S100A protein family [3]. Most AMPs are produced by epithelial and inflammatory cells, and AMP deficiency causes vulnerability to infection and inflammation [3]. In addition to their antimicrobial activity, AMPs are involved in modulating inflammation and activating the adaptive immunity [3].

Among the AMPs, PI3, and SLPI are antiproteases that inhibit serine proteases such as cathepsin G, chymotrypsin, and neutrophil elastase [4]. PI3 and SLPI are structurally related in that they have a whey acidic protein domain that is responsible for protease inhibition [5]. Activated neutrophils secrete numerous neutrophil serine proteases, including neutrophil elastase, proteinase 3, and cathepsin G [6]. Because neutrophil serine proteases have the potential to injure host tissues, serine protease inhibitors such as PI3 and SLPI are important for maintaining homeostasis by inhibiting proteolysis [6]. *PI3* and *SLPI* are expressed by neutrophils, macrophages, the decidua, and various epithelial cells in the respiratory, intestinal, and genital tracts [3]. The expression of *PI3* and *SLPI* is upregulated by alarm signals such as bacterial lipopolysaccharide, interleukin (IL)-1β, tumor necrosis factor (TNF)-α, and neutrophil elastase [4,7]. *PI3* expression is also induced by neutrophil elastase in alveolar epithelial cells [8].

The innate immune system in the female reproductive tract helps to protect the female from pathogens during the estrous cycle and during establishment and maintenance of pregnancy and parturition [9]. AMPs play an important role in the maintenance of pregnancy because infections are associated with various adverse pregnancy outcomes, including eclampsia, retarded fetal growth, recurrent miscarriage, and premature rupture of membranes [3]. The expression of *PI3* and *SLPI* in female reproductive tissues has been reported in humans and pigs. In humans, *SLPI* is expressed in the endometrium, decidua, and trophoblast [10]. The expression of *SLPI* is highest during the late secretory phase in the endometrial epithelium, and SLPI protein is localized to glandular epithelial cells in the endometrium and the decidua [10]. In pigs, *SLPI* is expressed in the endometrium, with increased level during mid to late pregnancy [11,12]. *PI3* expression is highest during menstruation, and PI3 protein is localized to endometrial neutrophils in humans [13]. Progesterone upregulates the expression of *SLPI*, but not *PI3*, in human breast epithelial cells [14]. *PI3* expression is upregulated by IL-1β and a combination of IL-1β and TNF-α in human primary endometrial epithelial cells [15].

The expression of *SLPI* in the endometrium throughout the estrous cycle and the expression and regulation of *PI3* in the endometrium during the estrous cycle and at the maternal–conceptus interface during pregnancy have not been fully studied in pigs. Therefore, we determined i) the expression of *PI3* and *SLPI* in the endometrium throughout the estrous cycle and pregnancy, in conceptus tissues during early pregnancy, and in chorioallantoic tissues during mid to late pregnancy; ii) localization of PI3 protein and *SLPI* mRNA in the endometrium; and iii) the effects of steroid hormones on *PI3* and *SLPI* expression in endometrial tissue explants.

## MATERIALS AND METHODS

### Animals and tissue preparation

All experimental procedures involving animals were conducted in accordance with the Guide for Care and Use of Research Animals in Teaching and Research and were approved by the Institutional Animal Care and Use Committees of Yonsei University (No. YWC-P120) and the National Institute of Animal Science (No. 2015-137). Sexually mature, crossbred gilts of similar age (6 to 8 months) and weight (100 to 120 kg) were randomly assigned to either cyclic or pregnant status. Gilts assigned to the pregnant status were artificially inseminated with fresh boar semen at the onset of estrus (Day 0) and 12 h later. The reproductive tracts of the gilts were obtained immediately after slaughter on Days 0 (onset of estrous behavior), 3, 6, 9, 12, 15, or 18 of the estrous cycle (21 days of cycle: Days 0 to 3, estrus; Days 3 to 6, metestrus; Days 6 to 15, diestrus; Days 15 to 0, proestrus) or Days 10, 12, 15, 30, 60, 90, or 114 of pregnancy (n = 3 to 6/d/status). Pregnancy was confirmed by the presence of apparently normal spherical to filamentous conceptuses in uterine flushings on Days 10, 12, and 15 and the presence of embryos and placenta on the later days of pregnancy [16]. Uterine flushings were obtained by introducing and recovering 25 mL of phosphate buffered saline (PBS; pH 7.4) into each uterine horn. Chorioallantoic tissues were obtained from Days 30, 60, 90, and 114 of pregnancy (n = 3 to 4/d). Endometrial tissues from prepubertal gilts (n = 8, approximately 6 months of age) that had not undergone an estrous cycle, with no corpus luteum formed, were obtained from a local slaughterhouse. Endometrial tissue, dissected free of myometrium, was collected from the middle portion of each uterine horn, snap-frozen in liquid nitrogen, and stored at −80°C prior to RNA extraction. For *in situ* hybridization and immunohistochemistry, cross-sections of endometrium were fixed in 4% paraformaldehyde in PBS (pH 7.4) for 24 h and then embedded in paraffin as previously described [17].

### Endometrial explant cultures

To determine the effects of the steroid hormones estradiol-17β (E2) and progesterone (P4) on the expression of *PI3* and *SLPI* mRNA in the endometrium, endometrial explant tissues from the prepubertal gilts were cultured as previously described [17]. The endometrium was dissected from the myometrium and placed into warm phenol red–free Dulbecco’s modified Eagle’s medium/F-12 culture medium (DMEM/F-12; Sigma, St. Louis, MO, USA) containing penicillin G (100 IU/mL) and streptomycin (0.1 mg/mL). The endometrium was minced with scalpel blades into small pieces (2 to 3 mm^3^), and aliquots of 500 mg were placed into T25 flasks with serum-free modified DMEM/F-12 containing 10 μg/mL insulin (Sigma, USA), 10 ng/mL transferrin (Sigma, USA), and 10 ng/mL hydrocortisone (Sigma, USA). Immediately after mincing, the endometrial explants were cultured in the presence of increasing doses of E2 (0, 5, 50, or 500 pg/mL; Sigma, USA) or P4 (0, 0.3, 3, or 30 ng/mL; Sigma, USA) for 24 h with rocking in an atmosphere of 5% CO_2_ in air at 37°C. The doses were chosen to encompass the full range of physiological levels of E2 and P4 [18]. The explant tissues were then harvested, and total RNA was extracted for use in real-time reverse transcription polymerase chain reaction (RT-PCR) to determine the effects of E2 and P4 on the expression of *PI3* and *SLPI* mRNA.

### Total RNA extraction, RT-PCR, and cloning of *PI3* and *SLPI* cDNA

Total RNA was extracted from endometrial and conceptus tissues using TRIzol reagent (Invitrogen Life Technology, Carlsbad, CA, USA) according to the manufacturer’s recommendations. The quantity of RNA was assessed spectrophotometrically, and the integrity of the RNA was validated following electrophoresis in 1% agarose gel. Four micrograms of total RNA from endometrial, conceptus, and chorioallantoic tissues were treated with DNase I (Promega, Madison, WI, USA) and reverse transcribed using SuperScript II reverse transcriptase (Invitrogen, USA) to obtain cDNA. The cDNA templates were then diluted 1:4 with sterile water and amplified by PCR using Taq polymerase (Takara Bio, Shiga, Japan). The final PCR reaction volume of 50 μL contained 3 μL of cDNA, 5 μL of 10× PCR buffer, 4 μL of dNTP mix (2.5 mM), 1 μL of each primer (20 μM), 0.3 μL of Taq polymerase (Takara Bio, Japan), and 36.7 μL of ddH_2_O. The PCR conditions, sequences of primer pairs for *PI3* and *SLPI*, and expected product sizes are listed in Table 1. The PCR products were separated on 2% agarose gels and visualized by ethidium bromide staining. The identity of each amplified PCR product was verified by sequence analysis after cloning into the pCRII vector (Invitrogen, USA).

### Quantitative real-time RT-PCR

The expression of *PI3* and *SLPI* mRNA in endometrial and chorioallantoic tissues was analyzed by real-time RT-PCR using an Applied Biosystems StepOnePlus system (Applied Biosystems, Foster City, CA, USA). Power SYBR Green PCR master mix (Applied Biosystems, USA) was used for the PCR reactions. The final reaction volume of 20 μL contained 2 μL of cDNA, 10 μL of 2× master mix, 2 μL of each primer (2 μM), and 4 μL of ddH_2_O. PCR was performed with an initial incubation at 95°C for 10 min, followed by 40 cycles of 15 s at 95°C and 30 s at 60°C. The sequences of primer pairs are listed in Table 1. The results are reported as expression relative to that detected on Day 0 of the estrous cycle in endometrial tissues, that on Day 30 of pregnancy in chorioallantoic tissues, or that detected in control explant tissues after normalization of the transcript amount to the geometric mean of endogenous ribosomal protein L7 (*RPL7*), ubiquitin B (*UBB*), and TATA binding protein (*TBP*) controls by the 2^−ΔΔCT^ method, as previously described [19].

### Immunohistochemistry

To determine the type(s) of cells expressing PI3 protein in the porcine endometrium, immunohistochemistry was performed. Uterine tissue sections (5 μm thick) were deparaffinized and rehydrated in an alcohol gradient. Tissue sections were washed with PBS with 0.1% (v/v) Tween-20 (PBST), and endogenous peroxidase activity was blocked with 0.5% (v/v) H_2_O_2_ in methanol for 30 min. Tissue sections were then blocked with 10% normal goat serum for 30 min at room temperature. Rabbit polyclonal anti-PI3 antibody (3 μg/mL; Proteintech, Chicago, IL, USA) was added, and sections were incubated overnight at 4°C in a humidified chamber. For each tissue tested, purified normal rabbit immunoglobulin G (IgG) was substituted for the primary antibody as a negative control. Tissue sections were washed intensively with PBST. Biotinylated goat anti-rabbit secondary antibody (1 μg/mL; Vector Laboratories, Burlingame, CA, USA) was added, and sections were incubated for 1 h at room temperature. Following washes with PBST, a streptavidin peroxidase conjugate (GBI Labs, Bothell, WA, USA) was added to the tissue sections, which were then incubated for 10 min at room temperature. The sections were washed with PBST, and aminoethyl carbazole substrate (Vector Laboratories, USA) was added to the tissue sections, which were then incubated for 20 min at room temperature. The tissue sections were washed in water, counterstained with Mayer’s hematoxylin, and coverslipped. Images were captured using an Eclipse TE2000-U microscope (Nikon, Seoul, Korea) and processed with Adobe Photoshop CS6 software (Adobe Systems, Seattle, WA, USA).

### Non-radioactive *in situ* hybridization

Nonradioactive *in situ* hybridization was performed to determine the localization of *SLPI* mRNA expression in the endometrium, as previously described with some modifications [17]. Tissue sections (5 μm thick) were rehydrated through successive baths of xylene, 100% ethanol, 95% ethanol, diethylpyrocarbonate (DEPC)-treated water, and DEPC-treated PBS. The sections were boiled in citrate buffer (pH 6.0) for 10 min. After being washed in DEPC-treated PBS, they were digested using 5 μg/mL Proteinase K (Sigma, USA) in TE (100 mM Tris-HCl, 50 mM ethylenediaminetetraacetic acid, pH 7.5) at 37°C. After post-fixation in 4% paraformaldehyde, the tissue sections were incubated twice for 15 min each in PBS containing 0.1% active DEPC and then equilibrated for 15 min in 5× saline sodium citrate (SSC). The sections were prehybridized for 2 h at 68°C in hybridization mix (50% formamide, 5× SSC, 500 μg/mL herring sperm DNA, 250 μg/mL yeast tRNA). Sense and antisense riboprobes for each gene were generated using partial cDNAs cloned into pCRII vectors by linearizing them with appropriate restriction enzymes and labeling them with digoxigenin (DIG)-UTP using a DIG RNA labeling kit (Roche, Indianapolis, IN, USA). The probes were denatured for 5 min at 80°C and added to the hybridization mix. The hybridization reaction was carried out overnight at 68°C. The prehybridization and hybridization reactions were performed in a box saturated with a 5× SSC 50% formamide solution to prevent evaporation, and no coverslips were used. After hybridization, the sections were washed for 30 min in 2× SSC at room temperature, 1 h in 2× SSC at 65°C, and 1 h in 0.1× SSC at 65°C. Probes bound to the section were detected immunologically using sheep anti-DIG Fab fragments covalently coupled to alkaline phosphatase and nitro blue tetrazolium chloride/5-bromo-4-chloro-3-indolyl phosphate (toluidine salt) as a chromogenic substrate, according to the manufacturer’s protocol (Roche, USA). The tissue sections were then washed in water and coverslipped. Images were captured using an Eclipse TE2000-U microscope (Nikon, Korea) and processed with Adobe Photoshop CS6 software (Adobe Systems, USA).

### Statistical analysis

Prior to the analysis, all data were tested for normality and homogeneity of variances. When necessary, log and square root transformations were performed. Data from the real-time RT-PCR for PI3 and SLPI expression were subjected to analysis of variance using the general linear models procedures of SAS (Cary, NC, USA). As sources of variation, the model included day, pregnancy status (cyclic or pregnant, Days 12 and 15 post-estrus), and their interactions to evaluate steady-state levels of *PI3* and *SLPI* mRNA. Data from the real-time RT-PCR performed to assess the effects of day of the estrous cycle (Days 0, 3, 6, 9, 12, 15, and 18) and pregnancy (Days 10, 12, 15, 30, 60, 90, and 114) in the endometrium and the effects of day of pregnancy in chorioallantoic tissues (Days 30, 60, 90, and 114) were analyzed using a least squares regression analysis. Data are presented as mean with standard error of the mean. A p-value <0.05 was considered significant, and p-values 0.05 to 0.10 were considered to indicate a trend toward significance.

## RESULTS

### Expression of *PI3* and *SLPI* in the endometrium during the estrous cycle and pregnancy

**Figure 1:**
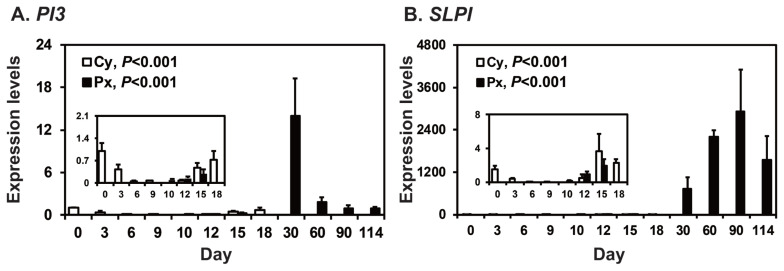
Expression of peptidase inhibitor 3 (*PI3*) and secretory leukocyte protease inhibitor (*SLPI*) in the endometrium during the estrous cycle and pregnancy. Endometrial tissue samples from cyclic (Cy) and pregnant gilts (Px) were analyzed by real-time reverse transcription polymerase chain reaction, and data are reported as expression relative to that detected on Day 0 of the estrous cycle after normalization of the transcript amount to the endogenous ribosomal protein L7, TATA binding protein, and ubiquitin B controls. Data are presented as means with standard errors.

To determine the patterns of *PI3* and *SLPI* expression in the endometrium throughout the estrous cycle and pregnancy in pigs, we measured their relative abundance using real-time RT-PCR (Figure 1). During the estrous cycle, the expression of *PI3* and *SLPI* in the endometrium changed, with the greatest levels in the proestrus phase (quadratic effect of day for *PI3* and *SLPI*, p<0.001). On Days 12 and 15 post-estrus, the expression of *PI3* was not affected by day, status, or day×status interaction, but the expression of *SLPI* was affected by day (p<0.05), though not by status or day×status interaction. During pregnancy, the steady-state levels of *PI3* and *SLPI* mRNA in the endometrium changed significantly, with the greatest levels on Day 30 of pregnancy for *PI3* mRNA (quadratic effect of day, p<0.001; Figure 1A) and higher levels during mid to late pregnancy than early pregnancy for *SLPI* mRNA (quadratic effect of day, p<0.001; Figure 1B).

### Localization of PI3 protein and *SLPI* mRNA in the endometrium on Days 12 and 15 of the estrous cycle and pregnancy

**Figure 2:**
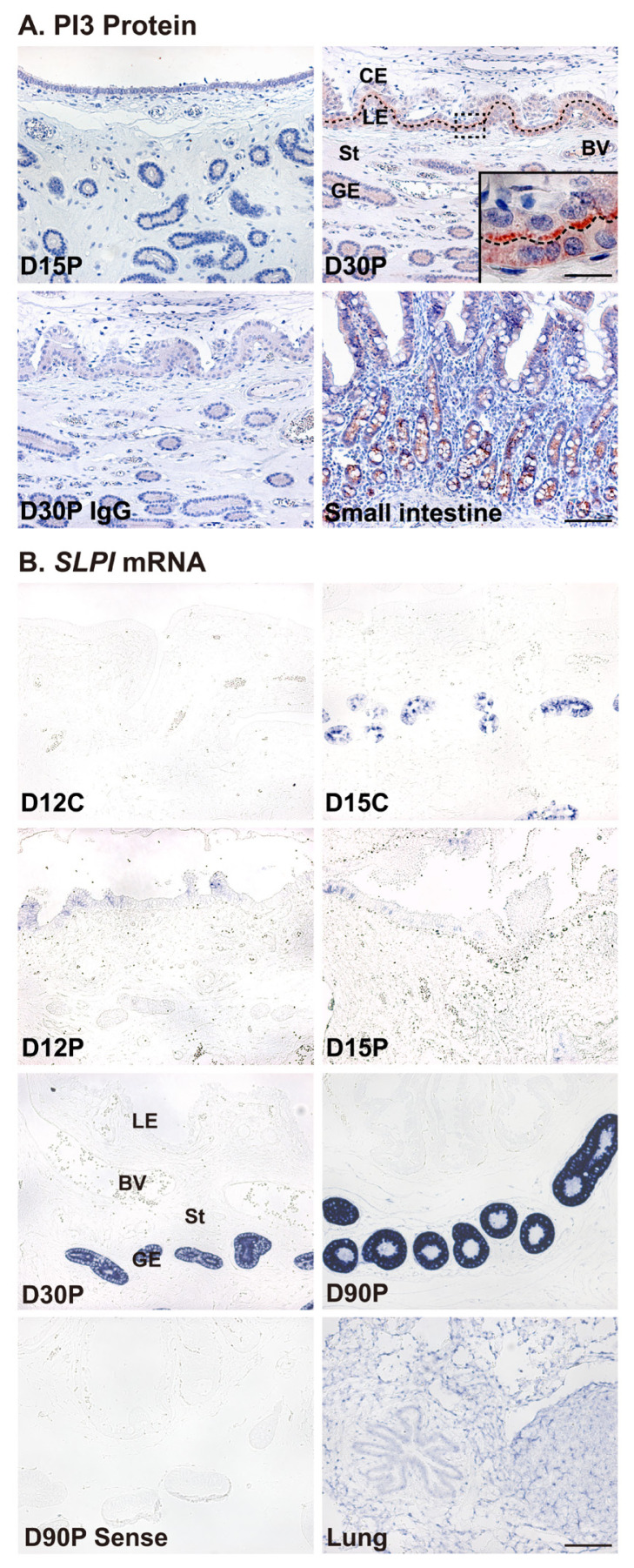
Localization of peptidase inhibitor 3 (*PI3*) and secretory leukocyte protease inhibitor (*SLPI*) mRNA in the endometrium during the estrous cycle and pregnancy. (A) Immunohistochemistry for the PI3 protein. Representative images from Days 15 and 30 of pregnancy are shown. A uterine section from Day 30 of pregnancy immunostained with normal immunoglobulin G (IgG) is shown as a negative control, and a tissue section from the small intestine is shown as a positive control. (B) *In situ* hybridization for *SLPI* mRNA. Representative images during the estrous cycle and pregnancy are shown. A uterine section from Day 30 of pregnancy hybridized with a digoxigenin-labeled sense *SLPI* cDNA probe (Sense) is shown as a negative control, and a tissue section from the lung is shown as a positive control. D, day; C, estrous cycle; P, pregnancy; LE, luminal epithelium; GE, glandular epithelium; CE, chorionic epithelium; St, stroma; BV, blood vessel. Bars = 100 μm, 20 μm in inset.

Localization of PI3 protein and *SLPI* mRNA was determined by immunohistochemistry and *in situ* hybridization analysis, respectively, in the endometrium during the estrous cycle and pregnancy in pigs. PI3 protein was localized primarily to luminal epithelial (LE) and glandular epithelial (GE) cells in the endometrium on Day 30 of pregnancy and to chorionic epithelial cells of the chorioallantoic membrane during mid to late pregnancy (Figure 2A). Immunostaining signals for PI3 protein were barely detectable in the endometrium during the estrous cycle (data not shown). *SLPI* mRNA was mainly localized to GE cells during mid to late pregnancy and to LE and GE cells at low levels during early pregnancy (Figure 2B). PI3 protein and *SLPI* mRNA were detected in the adult small intestine and adult lung, respectively and were used as positive controls.

### Expression of *PI3* and *SLPI* mRNA in conceptuses on Days 12 and 15 of pregnancy and chorioallantoic tissues on Day 30 to term pregnancy

**Figure 3:**
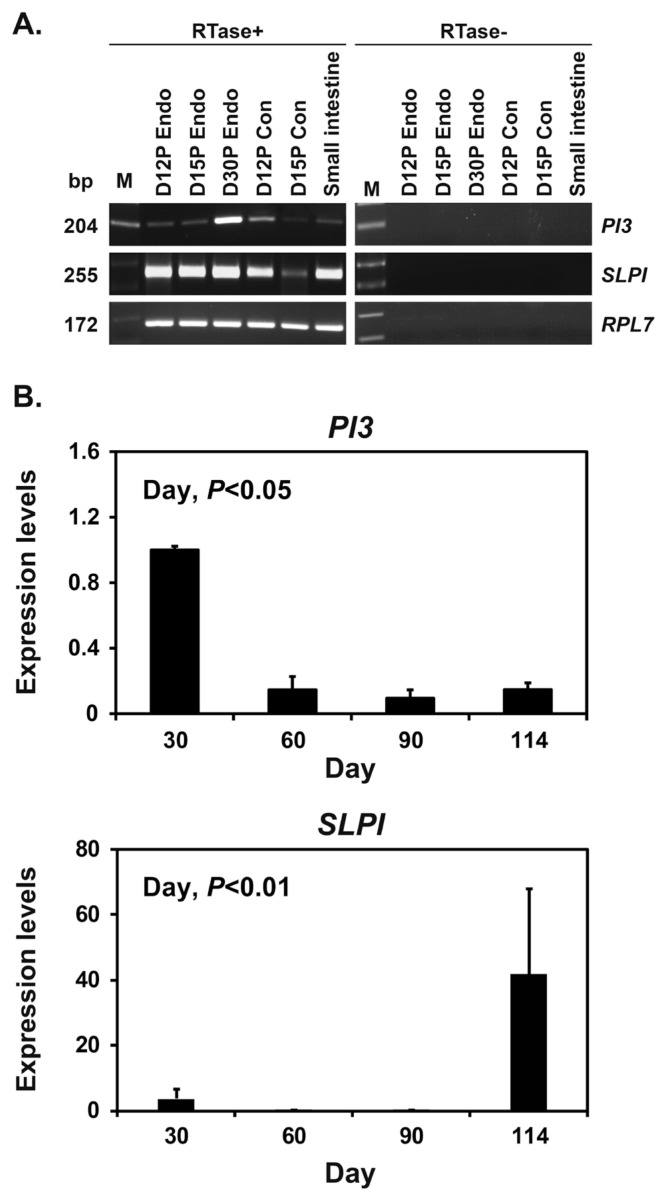
Expression of peptidase inhibitor 3 (*PI3*) and secretory leukocyte protease inhibitor (*SLPI*) in conceptuses from Days 12 and 15 of pregnancy and in chorioallantoic tissues during late pregnancy. (A) RT-PCR analysis of *PI3* and *SLPI* mRNA in conceptuses on Days 12 and 15 of pregnancy. Ribosomal protein L7 (*RPL7*) was used as a positive control in the polymerase chain reaction (PCR) analysis, and small intestine was used as a positive control. RTase +/−, with (+) or without (−) reverse transcriptase; M, molecular marker; D12 Endo, endometrium on Day 12 of pregnancy; D12 Con, Day 12 conceptus; D15 Con, Day 15 conceptus. (B) Real-time reverse transcription PCR analysis of the expression of *PI3* and *SLPI* mRNA in chorioallantoic tissues on Days 30, 60, 90, and 114 of pregnancy. Data are reported as expression relative to that detected on Day 30 of pregnancy after normalization of the transcript amount to the endogenous *RPL7*, TATA binding protein, and ubiquitin B controls, and data are presented as means with standard errors.

To determine whether conceptuses expressed *PI3* and *SLPI* mRNA during early pregnancy, we performed RT-PCR using cDNA from conceptuses on Days 12 and 15 of pregnancy. We found that *PI3* and *SLPI* mRNA were expressed in conceptuses on Days 12 and 15 of pregnancy, as well as in the small intestine tissue used as a positive control (Figure 3A). Real-time RT-PCR analysis was performed to determine whether the expression of *PI3* and *SLPI* changed in chorioallantoic tissues during mid to term pregnancy. The expression of *PI3* and *SLPI* mRNA in chorioallantoic tissues changed significantly during mid to term pregnancy, with decreasing and increasing levels, respectively, from Day 30 to term (quadratic effect of day, p<0.05 for *PI3* and p<0.01 for *SLPI*) (Figure 3B).

### Effects of E2 and P4 on *PI3* and *SLPI* expression in endometrial tissues

**Figure 4:**
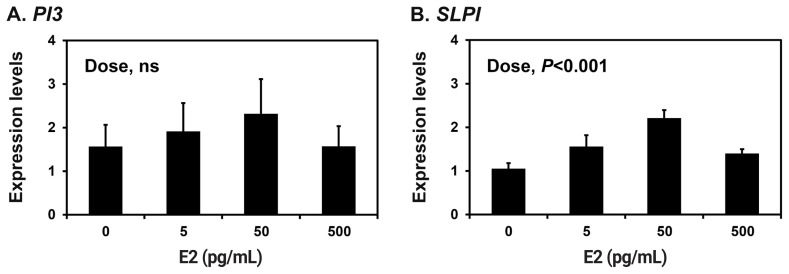
Effects of estradiol-17β (E2) on the expression of peptidase inhibitor 3 (*PI3*) and secretory leukocyte protease inhibitor (*SLPI*) in endometrial explant cultures. Endometrial explants from prepubertal gilts were cultured in the presence of increasing doses of E2 (0, 5, 50, or 500 pg/mL) at 37°C for 24 h. The abundance of mRNA expression shown in the real-time reverse transcription polymerase chain reaction analyses is relative to that of *PI3* and *SLPI* mRNA in the control group of endometrial explants (0 ng/mL) after normalization of the transcript amounts to ribosomal protein L7, TATA binding protein and ubiquitin B mRNA. Data are presented as least squares means with standard errors. For each treatment, experiments were performed in triplicate with endometria from eight gilts.

**Figure 5:**
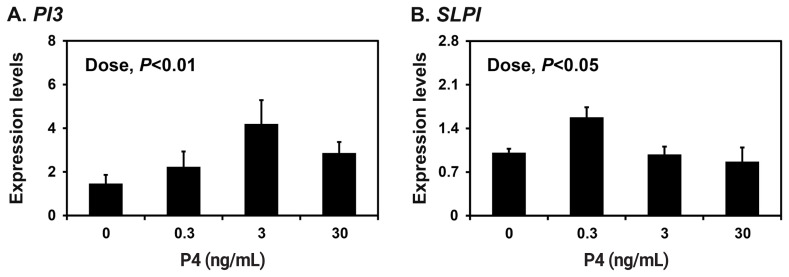
Effects of progesterone (P4) on the expression of peptidase inhibitor 3 (*PI3*) and secretory leukocyte protease inhibitor (*SLPI*) in endometrial explant cultures. Endometrial explants from prepubertal gilts were cultured in the presence of increasing doses of P4 (0, 0.3, 3, or 30 ng/mL) at 37°C for 24 h. The abundance of mRNA expression shown in the real-time reverse transcription polymerase chain reaction analyses is relative to that for *PI3* and *SLPI* mRNA in the control group of endometrial explants (0 ng/mL) after normalization of the transcript amounts to ribosomal protein L7, TATA binding protein, and ubiquitin B mRNA. Data are presented as least squares means with standard errors. For each treatment, experiments were performed in triplicate with endometria from eight gilts.

Because the endometrium is a major target of the ovarian steroid hormones E2 and P4 during the estrous cycle and pregnancy [18], we determined the effects of E2 and P4 on the expression of *PI3* and *SLPI* in endometrial explant tissues from prepubertal gilts that had not been exposed to cyclical ovarian hormones. The expression of *SLPI* (quadratic effect of dose, p<0.001), but not *PI3*, was upregulated by increasing doses of E2 in endometrial explants (Figure 4A and 4B). The expression of both *PI3* and *SLPI* was upregulated by increasing doses of P4 (quadratic effect of dose for *PI3*, p<0.05; cubic effect of dose for *SLPI*, p<0.05) (Figure 5A and 5B).

## DISCUSSION

The novel findings of this study in pigs are as follows: i) *PI3* and *SLPI* are expressed in the endometrium throughout the estrous cycle and pregnancy in an estrous- and pregnancy-stage-dependent manner; ii) PI3 protein is localized to the endometrial epithelia, with a strong signal intensity on Day 30 of pregnancy, and *SLPI* mRNA is localized to the endometrial epithelia, with a strong signal intensity in GE cells during mid to late pregnancy; iii) early stage conceptuses (Days 12 and 15) and chorioallantoic tissues from Day 30 to term pregnancy express *PI3* and *SLPI* mRNA; and iv) E2 increases the expression of *SLPI*, and P4 upregulates *PI3* and *SLPI* in endometrial explant tissues.

PI3 and SLPI act as antiproteases and AMPs and are ex pressed at mucosal surfaces in various tissues, such as the lung, small intestine, and endometrium [20]. It is well established that AMPs, including PI3 and SLPI, are expressed in the endometrium during the estrous cycle and pregnancy in various species, including cows, mice, and humans, to protect against potential pathogens [3,13,21]. In humans, the expression of *PI3* peaks during menstruation, and the expression of *SLPI* peaks during the late secretory phase of the menstrual cycle [10,15]. We previously reported that several AMPs, such as the cathelicidin family [22], the S100 family [23,24], and the beta-defensin family (Lee and Ka, unpublished data), are also differentially expressed in the endometrium during the estrous cycle and at the maternal–conceptus interface during pregnancy in pigs. In this study, we found that *PI3* and *SLPI* are expressed in the endometrium throughout the estrous cycle and pregnancy. Furthermore, the endometrial expression of *PI3* and *SLPI* changed depending on the stage of the estrous cycle and pregnancy; the expression of *PI3* and *SLPI* was highest at the estrus phase and at the proestrus phase, respectively, during the estrous cycle and on Day 30 and during the mid to late stage, respectively, during pregnancy. The expression of *SLPI* in the endometrium at the conceptus attachment sites during pregnancy was previously reported in pigs [11], but we have further determined the pattern of *SLPI* expression throughout the estrous cycle and pregnancy. Our results also show that *PI3* and *SLPI* are expressed in conceptus tissues during early pregnancy and in chorioallantoic tissues during mid to late pregnancy. Both PI3 and SLPI are known to have a broad spectrum of activity against Gram-positive and Gram-negative bacteria [3]. Because the stage of increased expression of *PI3* and *SLPI* during the estrous cycle corresponds with the estrogen-dominant period, we postulate that the increased *PI3* and *SLPI* at this stage of the cycle are involved in protecting the endometrium from possible bacterial contamination at mating. In addition, the levels of both *PI3* and *SLPI* in the endometrium were much higher during pregnancy than in the estrous cycle, and *PI3* and *SLPI* were expressed in conceptus and chorioallantoic tissues during pregnancy, which suggests that the antimicrobial activity of *PI3* and *SLPI* could play an important role in protecting the maternal–conceptus interface from microbial contamination to protect and maintain pregnancy.

The results of this study show that PI3 protein was localized to epithelial cells in both the endometrium and chorioallantoic tissues at the attachment sites, with strong signal intensity on Day 30 of pregnancy. It has already been shown that various AMPs, including PI3 and SLPI, are present in the epithelia of the endometrium and fetal membranes in other species, such as humans and cows [25], suggesting that AMP expression at the maternal–conceptus interface is common across many mammalian species. As reported for SLPI protein localization at the maternal–conceptus interface in pigs [26], the expression of *SLPI* mRNA was primarily localized to endometrial GE cells during mid to late pregnancy and to LE and GE cells at low levels during early pregnancy, suggesting the possibility of SLPI secretion into the maternal–conceptus interface to protect the conceptus attachment sites during pregnancy.

The endometrium is a major target of estrogen and pro gesterone during the estrous cycle and pregnancy [18], and the expression of *PI3* and *SLPI* changed during the estrous cycle and pregnancy in this study. Although the expression of *PI3* under the influence of steroid hormones is not well studied, the expression of *SLPI* is increased by estrogen in uterine epithelial cells and by progesterone in mammary epithelial cells in humans [27]. Therefore, we postulated that E2 and P4 might regulate endometrial *PI3* and *SLPI* expression. Indeed, the expression of *PI3* was upregulated by P4, and *SLPI* expression was dose-dependently increased by both E2 and P4 in endometrial explants. Our results also coincide with a previous report showing that estrogen and progesterone upregulate the expression of *SLPI* in porcine endometrial GE cells [28]. Because the expression of *SLPI* was highest during the estrus phases of the estrous cycle, when the plasma concentration of E2 is at its highest level [18], and during mid to late pregnancy, when chorioallantoic placentas produce both estrogen and progesterone in pigs [29], it is possible that ovarian estrogen is responsible for endometrial *SLPI* expression during the estrous cycle, and that estrogen and progesterone are together responsible for *SLPI* expression during mid to late pregnancy in pigs. Although *PI3* expression was highest at the estrus phase of the estrous cycle, E2 did not affect its expression, though P4 increased *PI3* expression in this study. In human endometrial epithelial cells, pro-inflammatory cytokines such as IL-1β and TNF-α are known to increase the expression of *PI3* [15]. In pigs, the pro-inflammatory cytokines IL-1β, IL-6, and TNF-α are expressed in the endometrium during the estrous cycle and pregnancy [30]. Thus, these data suggest that other factors, including pro-inflammatory cytokines, could be involved in the expression of PI3 during the estrogen-dominant period of the estrous cycle.

Trophoblasts are a rich source of proteases that induce degradation of the endometrial epithelium for trophoblast invasion. However, trophoblast invasion is controlled by protease inhibitors, and the degree of invasion determines the criteria for classifying placental types [31]. Pigs form a true epitheliochorial placenta, in which trophoblast invasion does not occur, so the endometrial epithelial cell layer remains intact until term during pregnancy [32]. Although PI3 and SLPI are well known AMPs found in various epithelial tissues that act in the innate immune response, they also act as protease inhibitors, blocking the activity of many enzymes, including cathepsin G, chymotrypsin, and neutrophil elastase [4]. It has been proposed that *SLPI* is expressed in the endometrium at high levels during late pregnancy in domestic animals that form epitheliochorial placentas, such as pigs, mares, and cows [33]. Thus, it is possible that the *PI3* and *SLPI* mRNA expressed at the maternal–conceptus interface not only protect the mother and fetus from infections through their antimicrobial activity, but also inhibit trophoblast invasion to enable the development and maintenance of the epitheliochorial placenta through their antiprotease activity.

In conclusion, the results of this study in pigs demonstrate that the serine protease inhibitors *PI3* and *SLPI* are differentially expressed in the endometrium during the estrous cycle and pregnancy and at the maternal–conceptus interface during pregnancy; *PI3* and *SLPI* expression was localized primarily to endometrial epithelial cells; and steroid hormones induced the expression of *PI3* and *SLPI* in endometrial tissues. Although the detailed functions of PI3 and SLPI need to be further determined, our findings indicate that the antimicrobial proteins PI3 and SLPI are expressed stage-specifically at the maternal–conceptus interface and might thus play important roles in both protecting maternal and conceptus tissues from potential pathogens and establishing the epitheliochorial placenta by regulating trophoblast invasion.

## Figures and Tables

**Table 1:** Summary of primer sequences for real-time RT-PCR and RT-PCR and expected product sizes

Primer	Sequence of forward (F) and reverse (R) primers (5′ → 3′)	Annealing temperature (°C)	Product size	GenBank accession no. (bp)
For RT-PCR				
*PI3*	F: TCAAGGGTCAAGATCCATTC	60	204	NM_001123215.1
	R: AGCCTTCACAGCACTTCTTG			
*SLPI*	F: CTAGAAAAATTGTCCAGTGCCTTAG	60	255	NM_213870.1
	R: ACATGCTCTTGCAGCATTTTAAGTC			
*RPL7*	F: AAG CCA AGC ACT ATC ACA AGG AAT ACA	60	172	NM_001113217
	R: TGC AAC ACC TTT CTG ACC TTT GG			
For real-time PCR				
*PI3*	F: TCAAGGGTCAAGATCCATTC	60	204	NM_001123215.1
	R: AGCCTTCACAGCACTTCTTG			
*SLPI*	F: CTAGAAAAATTGTCCAGTGCCTTAG	60	255	NM_213870.1
	R: ACATGCTCTTGCAGCATTTTAAGTC			
*RPL7*	F: AAG CCA AGC ACT ATC ACA AGG AAT ACA	60	172	NM_001113217
	R: TGC AAC ACC TTT CTG ACC TTT GG			
*UBB*	F: GCA TTG TTG GCG GTT TCG	60	65	NM_001105309.1
	R: AGA CGC TGT GAA GCC AAT CA			
*TBP*	F: AAC AGT TCA GTA GTT ATG AGC CAG A	60	153	DQ845178.1
	R: AGA TGT TCT CAA ACG CTT CG			
For *in situ* hybridization				
*SLPI*	F: CTAGAAAAATTGTCCAGTGCCTTAG	60	255	NM_213870.1
	R: ACATGCTCTTGCAGCATTTTAAGTC			

RT-PCR, reverse transcription polymerase chain reaction; *PI3*, peptidase inhibitor 3; *SLPI*, secretory leukocyte protease inhibitor; *RPL7*, ribosomal protein L7; *UBB*, ubiquitin B; *TBP*, TATA binding protein.
